# Children with rare diseases, is it true that they have an enhanced anesthesiological risk?

**DOI:** 10.1186/1824-7288-41-S2-A63

**Published:** 2015-09-30

**Authors:** Maria Sammartino, Fabio Sbaraglia, Francesco A Idone

**Affiliations:** 1 Department of Anesthesia and Intensive Care, Catholic University of Sacred Heart, Rome, Italy

## 

The anesthesiological approach to rare diseases (RD) is particularly challenging for providers. The high number of pathologies and the scarcity of clinical reports impedes to arrange a pool of dedicated anaesthesiologists. When facing RD, the risk linked to unaware malpractice is often due to lack of experience, mostly of urgent/emergent procedures [1].

It is probably impossible to know all relevant aspects in more than 8000 pathologies that affect approximately 6-8% of the population [2]. In order to reduce anesthesiological risk, it is mandatory to develop a systematic approach.

1. First and foremost, let's take advantage of previous experiences. There are specific search engines available online [http://www.orphananesthesia.eu; http://www.orpha.net; http://www.omim.org

], which briefly summarize the knowledge about RDs. Furthermore, for major pathologies there are national and international main centres providing medical counselling.

As a whole, we can attribute the anesthesiological risk to four cohorts.

Careful evaluation should be dedicated to current systemic impairments [3]. Children affected by cystic fibrosis need special care, due to specific organ failure. In some types of porphyrias it is mandatory to achieve a good control on pain, anxiety and nausea, because stressful events could trigger a severe crisis.

In some cases, pathological processes can affect pharmacodynamics causing abnormal responses to drugs, such as the enhancement of curarization in myasthenic patients, or a real adverse event like the risk of Malignant Hyperthermia associated with Wolf-Hirschhorn Syndrome [4] or Arthrogryposis.

A third item to investigate is whether anesthetic drugs can interact with specific concurrent therapies. There are some suggestions that the previous therapy with eculizumab, a new monoclonal antibody for the treatment of paroxysmal nocturnal haemoglobinuria, predisposes to haemolytic crisis in occurrence of general anesthesia.

The last cohort, but the most frequent, concerns the difficult management of airways. Craniofacial malformations can represent a very big deal due to mandible hypoplasia such as Pierre Robin syndrome or Treacher-Collins syndrome; hemifacial microsomia such as Goldenhar syndrome; midface hypoplasia as Apert syndrome [5], Crouzon syndrome, Pfeiffer syndrome and macroglossia such as Hurler's syndrome, Hunter's syndrome, or weakness of atlanto-occipital joint such as Downs syndrome, Ehlers-Danlos syndrome type IV or osteogenesis imperfecta. Tracheal intubation should indeed be avoided in syndromes as epidermolysis bullosa to prevent severe tracheal lesions.

In conclusion, besides patients with a clear diagnosis, sometimes a simple pathology can hide unknown RDs [6]. For this reason the pre-anesthesiological evaluation is crucial, more than ever in syndromic children, to assess the risk before undergoing general anesthesia or sedation. This allows the best strategy to work safely to be put in place and to improve outcome.
